# Perioperative management of septic peritonitis in small animals: A review

**DOI:** 10.1111/vsu.70051

**Published:** 2025-11-03

**Authors:** Shana K. O'Marra, Bonnie G. Campbell

**Affiliations:** ^1^ Washington State University Pullman Washington USA

## Abstract

**Background:**

Septic peritonitis (SP) is a complex, life‐threatening disease, driven by peritoneal inflammation and microbial contamination, requiring timely and dynamic perioperative management.

**Aims:**

The aim of this review was to synthesize current knowledge on the perioperative management of SP in dogs and cats.

**Conclusions:**

Evidence‐based strategies for initial stabilization include fluid resuscitation with balanced crystalloids, asopressors, and antimicrobial therapy targeting polymicrobial infections. Anesthetic management should prioritize hemodynamic stability and a multimodal approach to analgesia. Postoperative management should include early enteral nutrition (which is associated with increased survival) and monitoring and treatment of coagulation derangements. Patients should be closely monitored for recurrent SP after surgery, which is associated with high mortality. Evidence for risk factors of dehiscence such as hypoalbuminemia and interoperative hypotension is inconsistently found in studies. Other potential complications include hospital acquired infection and intra‐abdominal hypertension.

**Implications:**

There is significant variation in the treatment approach for small animals with SP, likely due to gaps in evidence. Reported survival rates vary widely between studies due to diverse and inconsistent study populations, highlighting the need for further research to optimize care in veterinary patients.

## 1 INTRODUCTION

The peritoneum lines the abdominal cavity and covers organs and mesenteries. Its cells maintain peritoneal homeostasis, regulating movement of molecules and cells across the peritoneum and keeping inflammatory and immune‐mediated processes in check. Insults like trauma (including surgery), inflammation, ischemia, bacteria, and endotoxins stimulate a mesothelial‐to‐mesenchymal transition. These transformed cells orchestrate a multifaceted set of pro‐ and anti‐inflammatory and immunomodulatory processes designed to restore balance.^1^, ^2^, ^3^, ^4^, ^5^, ^6^, ^7^ The complexity of these processes helps one understand how peritonitis can sometimes rapidly progress beyond the point where balance can be restored.

Septic peritonitis (SP) is characterized by peritoneal inflammation and microbial contamination. It is associated with high morbidity and mortality, with survival rates even lower for SP occurring after surgery for an initial SP. Evidence‐based perioperative management of SP centers on timely restoration of hemodynamic stability, adequate analgesia, empiric broad‐spectrum antimicrobials with appropriate de‐escalation, early postoperative enteral nutrition, and vigilant postoperative monitoring and intervention for conditions like recurrence of SP after surgery for SP, hospital‐acquired infection, and intra‐abdominal hypertension. This review brings together current insights into perioperative therapeutic strategies in SP to improve outcome in veterinary patients. Initial diagnosis and intraoperative source‐control strategies are addressed in a companion manuscript.^8^

Pertinent papers were collected via a PubMed search for “septic peritonitis,” “septic effusion” and “peritonitis” AND “Dog” OR “Cat” OR “Veterinary” from the last 15 years. Additional sources were identified via “cited by” or “similar articles,” from relevant manuscripts, or additional subtopic searches. The authors focused on case series where the study period extended into the last 15 years.

## 2 SEPTIC PERITONITIS CLASSIFICATION

Septic peritonitis is classified as primary, secondary, or tertiary. Primary SP (PSP) involves peritoneal infection with no intra‐abdominal source. It is most common in people with free abdominal fluid (usually from cirrhosis or peritoneal dialysis).^9^, ^10^, ^11^ Human PSP is usually monomicrobial and managed with antimicrobials and supportive care, not surgery.^9^, ^10^, ^11^ In dogs and cats with PSP, mono‐ and multimicrobial infections, and Gram‐positive and ‐negative organisms, have been reported.^12^, ^13^, ^14^, ^15^ While non‐surgical management may be adequate, it is difficult to identify PSP short of a negative surgical explore.

Secondary SP (SSP), by far the most common in veterinary patients, arises from bacteria introduced via a penetrating injury or an intra‐abdominal source, most commonly the gastrointestinal tract (Table 1).^11^, ^12^ It is typically polymicrobial and requires surgical source control. Secondary SP is the main focus of this review and its companion study.^8^

**TABLE 1:** Survival rates and findings in dogs and cats having surgery for septic peritonitis.

Study years^a^, type	Main inclusion criteria in addition to surgery for SP	Most common cause of SP	Overall survival rate^b^	Impact of selected factors^c^ on survival
2000–11 R^16^	Dogs with surgery for RecSP	90.3% GI	43.9% (18/41)	NSD if: Open abdomen vs. closed abdomen +/− closed suction drain Foreign body was cause of sp Appropriate vs. inappropriate empirical antimicrobials
2002–11 R^17^	Dogs (44) and cats (11) with SP of GI origin	100% GI	36.4% (20/55)	NSD if: Pyloric vs. non‐pyloric GI perforation Did vs. did not receive prior anti‐inflammatory drugs Primary closure with vs. without closed‐suction drain
2003–11 R^18^	Dogs	74% GI	57.0% (49/86)	92% (34/37) if abdominal infection^d^ 45% (14/31) if severe sepsis^d^ 6% (1/18) if septic shock^d^ NSD if appropriate vs. inappropriate empirical antimicrobials
2004–11 R^19^	Dogs surviving ≥24 h postoperatively	75% GI	76.7% (43/56)	81.4% (35/43) with EN^d^ 46.2% (6/13) without EN^d^
2007–11 R^20^	Dog receiving intraoperative opioid, or opioid+lidocaine	>75% GI	73.3% (55/75) at 48 h postoperatively	60.6% (20/33) with opioid^d^ 83.3% (35/42) with opioid+lidocaine^d^
2007–12 R^21^	Dogs with ≥1 lactate measurement	89% GI	64% (53/83)	Severity scores were signficantly higher in non‐survivors
2000–13 R^22^	Dogs	62.4% GI	66.7% (116/174)	84.3% (113/134) if no RecSP^e^ 42.9% (3/7) if RecSP and ≥1 relaparotomy^e^ 0% (0/25) if euthanized intraoperatively 0% (0/8) if euthanized for RecSP without relaparotomy
2012–14 P^23^	Dogs with SP of GI origin and closed suction drain postoperatively	100% GI	88.5% (23/26)	100% (23/23) if no RecSP^e^ 0% (0/3) if RecSP^e^
2012–14 P^24^	Dogs randomized to VAC (8) or open abdomen (8)	75% GI	81% (13/16)	NSD if VAC vs. open abdomen
2002–15 R^13^	Cats	49.9% GI	69.9% (58/83)	80% if appropriate empirical antimicrobials^d^ 55% if inappropriate empirical antimicrobials^d^ NSD for primary vs. secondary SP
2007–15 R^25^	Dogs	80% GI	60% (33/55)	NSD based on time from admittance to surgery
2011–15 R^26^	Dogs (29) or cats (6) with empirical antimicrobials and pre‐ and post‐lavage cultures	68.8% GI	74.3% (26/35)	NSD if: Dog vs. cat Appropriate vs. inappropriate empirical antimicrobials
2011–15 P^27^	Dogs with culture pre‐and post‐lavage	65% GI	87.5% (35/40)	NSD if appropriate (*n* = 39) vs. inappropriate (*n* = 1) empirical antimicrobials
2006–16 R^28^	Dogs with placement of gastrostomy tube	76.7% GI	51% (22/43)	Descriptive study
2009–16 R^29^	Dogs with placement of gastrostomy tube	75% GI	75% (18/24)	Non‐survivors took significantly longer to eat voluntarily (if at all)
2012–17 R^30^	Dogs	57.1% non‐neoplastic GI	75% (42/56)	Non‐survivors had significantly higher severity scores
2008–18 R^31^	Cats	51% GI	66.3% (63/95)	Non‐survivors had signficantly higher incidence of intraoperative hypotension NSD if appropriate vs. inappropriate empirical antimicrobials
2011–18 R^32^	Dogs with >2 SIRS criteria	Not reported	65% (93/143)	Non‐survivors had signficantly lower blood pressure and significantly higher severity score
2009–19 R^33^	Dogs surviving surgery	63% GI	72% (85/115)	77% if no closed suction drain (*n* = 93)^d^ 53% if closed suction drain (*n* = 22)^d^
2003–20 R^34^	Dogs surviving surgery for SP due to GI or biliary leakage	94.8% GI	75.9% (44/58)	NSD based on: Location of leak along GI Bacteria cultured
2004–20 R^35^	Dogs	54.9% GI	74.3% (84/113)	Non‐survivors had signficantly higher incidence of liver injury, acute kidney injury, and intra‐ or postoperative hypotension
2010–20 R^36^	Dogs	67.4% GI	70.9% (61/86)	Non‐survivors had signficantly higher severity scores NSD for neoplastic (*n* = 74) vs. non‐neoplastic (*n* = 12) cause
2014–21 R^37^	Dogs (6) and cat (1) with intraperitoneal grass awn	100% omental abscess	100% (7/7) (≥2‐month follow‐up)	Descriptive study

Abbreviations: EN, enteral nutrition; GI, gastrointestinal; NSD, no significant difference; P, prospective study; R, retrospective study; RecSP, recurrent SP; SIRS, systemic inflammatory response syndrome; SP, septic peritonitis; VAC, vacuum assisted closure.

^a^ Only studies looking at patient populations that extended at least into the last 15 years are included.

^b^ Survival is to discharge unless otherwise noted.

^c^ Biochemical findings are reported in a companion paper.^8^

^d^ Significant difference between paired items in the study.

^e^ Significance not reported.

Tertiary SP (TSP) is has not been conclusively defined in veterinary species.^16^ Definitions for TSP in people vary, but emphasize recurrence of SP despite successful treatment of primary or secondary SP.^10^, ^38^, ^39^, ^40^ In people, TSP often occurs within a week of surgery,^16^ and is linked to a compromised immune system that cannot prevent bacteria from taking advantage of residual peritoneal inflammation.^10^, ^41^ Treatment includes short‐duration antimicrobials if there is infection (vs. colonization) and supportive therapies, not surgery, although surgery may be needed to rule‐out SSP from an intra‐abdominal source such as anastomosis dehiscence.^40^, ^41^, ^42^ Multi‐resistant organisms and multiorgan failure frequently develop.^41^

## 3 FLUID RESUSCITATION

Septic patients often suffer from intravascular volume depletion, decreased vasomotor tone, redistribution of blood flow, and microcirculatory dysfunction. Transient myocardial depression has been documented in up to 50%–60% of septic people,^43^ and in dogs suffering from viral and bacterial sepsis.^44^, ^45^, ^46^, ^47^, ^48^, ^49^, ^50^ Hypo‐ or hypervolemia, vasomotor tone and endothelial glycocalyx integrity impact the distribution of crystalloids and colloids into intravascular and interstitial spaces.^51^

The 2021 Surviving Sepsis Guidelines^52^ (SSG) recommend immediate fluid resuscitation with balanced crystalloids for sepsis‐induced hypoperfusion or septic shock in people. At least 30 mL/kg are recommended within the first 3 h; however, controversy exists regarding the risk of fluid overload with this approach.^53^, ^54^ Early adequate fluid resuscitation followed by late conservative fluid management appears to be ideal in people with sepsis.^55^, ^56^

Consensus guidelines are lacking for dogs and cats with SP. It is reasonable to assume some degree of volume depletion, and resuscitation with a balanced crystalloid is warranted when perfusion deficits are noted. In people, a passive leg raise briefly increases cardiac preload to predict fluid responsiveness; this method is not feasible in veterinary patients. Instead, clinicians can perform a fluid challenge (administer a 3–5 mL/kg bolus rapidly) and monitor for hemodynamic improvement.^57^ When cardiac output and stroke volume cannot be easily measured, improved capillary refill time, shock index (heart rate ÷ systolic blood pressure), blood pressure, heart rate and mentation are surrogate markers of hemodynamic improvement.^57^, ^58^ A positive response should prompt a larger bolus of 10–20 mL/kg.^57^, ^59^, ^60^

A drawback to the fluid challenge is that lack of volume responsiveness is not evident until after bolus administration.^59^ The caudal vena cava collapsibility index (CVCCI) is a dynamic indicator of fluid‐responsiveness. It can be measured via ultrasound in awake, spontaneously breathing patients, using the techniques described by Darnis et al,^61^ to guide resuscitation.^57^ A study in critically ill dogs found a CVCCI >26.7% predicted fluid‐responsiveness with 100% sensitivity and 83.3% specificity.^62^

**FIGURE 1:**
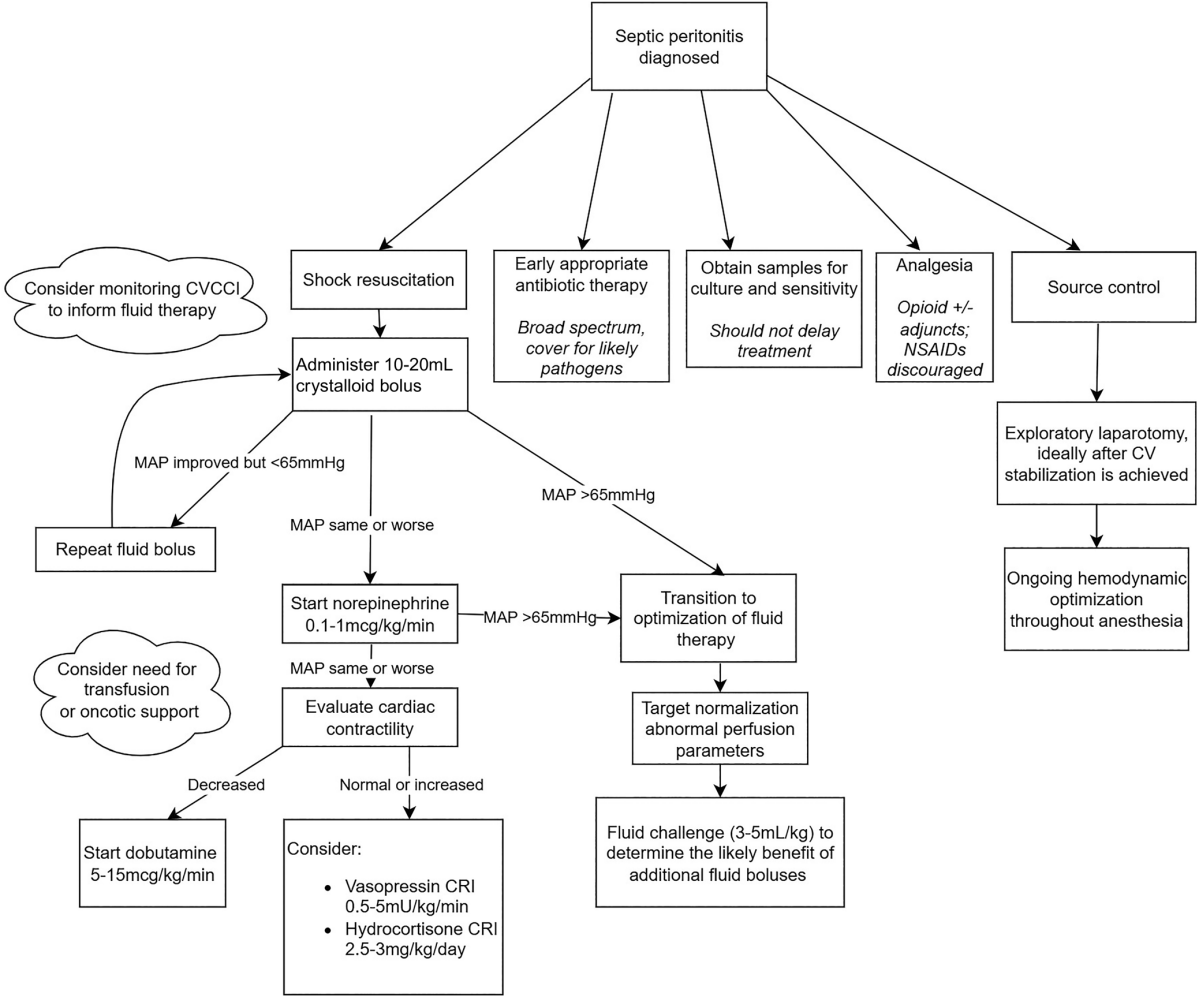
Initial approach to stabilization of the septic peritonitis patient. Initial diagnosis and intraoperative source‐control strategies are addressed in a companion manuscript.^8^ CRI, constant rate infusion; CV, cardiovascular; CVCCI, caudal vena cava collapsibility index; MAP, mean arterial blood pressure; NSAID, non‐steroidal anti‐inflammatory.

If hypotension (mean arterial pressure [MAP] <65 mmHg) persists despite intravascular repletion, vasopressors are recommended to counter the vasoplegia common with sepsis.^63^ Norepinephrine (0.1–1 mcg/kg/min) is the first‐line vasopressor in septic people,^64^ dogs, and cats.^59^, ^65^ If adequate MAP is not achieved with norepinephrine alone, vasopressin (0.5–5 mU/kg/min) can be added.^52^, ^65^ If hypotension remains refractory to pressor therapy, corticosteroids are suggested to restore catecholamine responsiveness.^52^ For patients with persistently low cardiac output, dobutamine (5–15 mcg/kg/min) is indicated.^52^, ^59^ A suggested algorithm for initial stabilization is presented in Figure 1.

Once intravascular volume is replenished, fluid overload must be avoided. In people, fluid overload is associated with ileus, intra‐abdominal hypertension, organ dysfunction, delayed healing, and increased mortality in sepsis.^66^, ^67^, ^68^ Dogs with fluid overload had longer length of hospitalization and decreased survival in one retrospective study.^69^ Frequent assessment of hydration, urine and drain output, and cumulative fluid balance, with vigilance for signs of serous nasal discharge, chemosis, new effusions and peripheral or pulmonary edema allow early detection of fluid overload. Point of care ultrasound may reveal new cavitary effusions, enlargement of the left atrium or caudal vena cava, and decreased CVCCI.^57^, ^59^

Although a balanced, isotonic crystalloid solution is recommended for initial resuscitation of septic shock, many septic human and veterinary patients are hypoalbuminemic,^70^, ^71^ raising concern for decreased plasma oncotic pressure. The SSG strongly recommends against using hydroxyethyl starches (HES) for resuscitation in people due to the absence of clear benefit and higher risk of needing renal replacement therapy or death.^72^ Instead, in septic people receiving large volumes of crystalloids, the SSG weakly recommend albumin.^52^

Studies evaluating acute kidney injury (AKI) at recommended doses of HES in veterinary species yield mixed results. Similar to people, tubular accumulation of HES occurred in critically ill dogs receiving 6% HES 130/0.4.^73^ Some clinical canine studies showed increased risk of renal injury or mortality with various hydroxyethyl starches,^74^, ^75^ while others found no evidence of harm.^76^, ^77^, ^78^, ^79^ A single study in a population of critically ill, non‐azotemic cats showed no association between HES‐130/0.4 dose or infusion duration and AKI.^80^

In vitro studies demonstrated hypocoagulability beyond the expected dilutional effects of HES in dogs and cats.^81^, ^82^, ^83^, ^84^, ^85^, ^86^, ^87^, ^88^ In vivo studies demonstrated HES‐induced hypocoagulability during hemorrhage^79^ and systemic inflammation.^89^ Two clinical studies demonstrated hypocoagulability in healthy dogs^90^ and dogs with hemoperitoneum^91^ receiving HES, while others found no effect in healthy dogs^92^ or across diverse diseases.^77^, ^93^, ^94^ Use of HES in septic veterinary patients remains controversial and should be avoided during initial resuscitation. If considered for oncotic support in select cases, dose and duration of administration should be limited to reduce risk of harm.^95^

Albumin administration was associated with improved survival in people with septic shock in a subgroup analysis of the Albumin Italian Outcome Sepsis (ALBIOS) trial.^96^, ^97^ In dogs with SP, human serum albumin (HSA) increased albumin levels and colloid osmotic pressure (COP) with no survival benefit.^98^ Human serum albumin use in dogs has been associated with serious to fatal immediate and delayed hypersensitivity reactions,^99^, ^100^, ^101^ although risk may be lower in critically ill patients.^102^, ^103^ Dogs develop anti‐HSA antibodies after transfusion, precluding repeated administration.^99^, ^104^ Canine albumin (CA) may pose less risk of transfusion reaction in dogs, and appears to be effective,^105^ although availability is limited. Albumin, COP, and blood pressure increased shortly after CA administration in septic dogs, with no delayed hypersensitivity reactions; however, albumin increase only lasted 24 h.^105^ This study was not powered to detect mortality differences.

Plasma products have not generally been recommended for hypoalbuminemia due to cost, risk of transfusion reaction, and large volume required.^105^, ^106^ However, two recent studies report widespread use of plasma transfusion to treat hypotension or provide oncotic support in hypoalbuminemic veterinary patients,^107^, ^108^ with a low incidence of transfusion reaction.^107^ Limited data in rats and people suggest fresh frozen plasma (FFP) may mitigate endothelial glycocalyx shedding.^109^, ^110^, ^111^, ^112^ Cryopoor plasma (cryoprecipitate supernatant) has a higher albumin concentration (mg/dL) than FFP and has been used in dogs with SP.^113^, ^114^ Advantages include smaller transfusion volumes and lower cost.^114^

## 4 ANTIMICROBIAL THERAPY

Early appropriate antimicrobial therapy improves survival in human sepsis.^52^ The SSG strongly recommend immediate antimicrobial administration for adults with possible septic shock or a high likelihood of sepsis.^52^ Collection of culture samples should not delay treatment.

The impact of early appropriate antimicrobials in veterinary medicine is unclear. While antimicrobial administration within 1 h of sepsis diagnosis is feasible,^115^ retrospective canine studies have shown no survival benefit with appropriate, timely antibiotic therapy (Table 1).^16^, ^18^, ^26^, ^27^, ^31^, ^115^, ^116^ In contrast, cats with septic peritonitis receiving appropriate empirical antimicrobials were four times more likely to survive.^13^ Early antimicrobial therapy remains prudent pending further research (Figure 1).

### 4.1 Antibacterial drugs

Gram‐negative bacteria are the most common isolate in dogs and cats with SP, although Gram‐positive and polymicrobial infections are also common.^63^ Various antimicrobial strategies have been described in veterinary medicine, including amikacin or a third generation cephalosporin combined with clindamycin, ampicillin combined with a fluoroquinolone, single agent third generation cephalosporin, the addition of metronidazole to broaden anaerobic coverage, and many others.^18^, ^115^, ^117^ Antimicrobial selection should be broad spectrum, targeted to the source, and adjusted for local resistance patterns. For example, where there is a low incidence of antimicrobial resistance, ampicillin and enrofloxacin would be a reasonable first choice for a patient with jejunal perforation and no recent hospitalization or antibiotic administration.

Enteric colonization with multidrug resistant bacteria is common in dogs receiving antimicrobials or with prolonged (>3 days) intensive care unit (ICU) hospitalization.^118^ Antimicrobial administration >7 days in critically ill dogs was associated with antimicrobial resistant *E. coli* persisting for up to 60 days.^119^ A meta‐analysis of studies evaluating the enteric colonization of dogs with resistant *Enterobacteriales* found raw diet, antimicrobial use and hospitalization were risk factors in this diverse population of healthy and ill dogs.^120^

In dogs with SP, abdominal surgery and antimicrobial administration in the 30 days prior to admission were each associated with inappropriate empiric antimicrobial therapy.^18^ As enteric flora cause many hospital‐acquired infections, length of hospitalization, diet and antimicrobial‐administration histories should influence initial empiric choices. For example, a patient who has been receiving enrofloxacin for the 2 weeks prior to the development of SP should prompt the clinician to consider a non‐fluoroquinolone such as a third‐generation cephalosporin or aminoglycoside for Gram‐negative coverage.

Patient factors such as renal and hepatic function should also be considered. In septic people, failure to reach target antimicrobial levels is common, and associated with higher mortality.^121^ Increased renal blood flow in high cardiac output states increases clearance of renally excreted drugs,^122^ while renal and hepatic impairment may decrease drug clearance.^123^ Septic patients may also have altered volume of distribution.^124^ Therapeutic drug monitoring has been suggested to minimize the risk of subtherapeutic antimicrobial levels, but did not improve sequential organ failure assessment (SOFA) scores in septic people.^121^

One strategy to maximize the efficacy of time‐dependent antimicrobials is to deliver prolonged or constant rate infusions (CRI). A meta‐analysis of studies in people with sepsis or septic shock suggested reduced mortality with long infusions of beta lactam antimicrobials,^125^ but the recent Beta‐Lactam Infusion Group (BLING) III trial found no difference in 90 day survival between continuous and intermittent (30 min) β‐lactam administration.^126^ Meropenem CRIs achieve target concentrations in people more consistently than prolonged (3 h) infusions.^127^ In dogs with septic peritonitis, CRI of ampicillin‐sulbactam achieved 100% time above minimum inhibitory concentration (MIC) for all studied breakpoints, however intermittent dosing also achieved 100% time above MIC for breakpoints up to 1.25 μg/L.^128^

The optimal duration of antimicrobial therapy in SP is unknown. Human studies suggest that longer courses (>7 days) increased risk of antimicrobial resistance without improved survival.^129^ Reported median duration in veterinary patients septic from a variety of causes is 11 days.^130^ Antimicrobial therapy should be de‐escalated based on culture and sensitivity results. If there is suspicion for hospital‐acquired infection, new cultures should be obtained to guide further antimicrobial therapy.^130^

### 4.2 Antifungal drugs

Fungal sepsis is associated with poor outcomes in people, and response to treatment is unreliable.^131^, ^132^, ^133^ *Candida* peritonitis has been rarely reported in dogs.^134^, ^135^, ^136^ *Candida spp.* were first identified on cytologic evaluation of peritoneal effusion in most cases.^134^, ^135^ *Candida spp.* grow well on blood agar and may be found on routine antimicrobial culture.^135^ All reported canine cases were cultured positive for *Candida,* and most were treated with fluconazole IV or orally. Successful outcome was described in 3/7 dogs.^134^, ^135^, ^136^ Based on canine pharmacokinetics, a 5 mg/kg daily dose of fluconazole may be insufficient.^135^ The authors suggest that dogs with *Candida spp.* identified on antimicrobial culture receive treatment with fluconazole at 10 mg/kg every 12 h IV, transitioning to oral administration when tolerated,^137^ recognizing that robust data to support treatment are lacking. Optimal duration is unknown; human guidelines recommend that duration of therapy be determined by adequacy of source control and clinical response.^138^ To the authors' knowledge, *Candida* peritonitis has not been reported in cats.

## 5 ANALGESIA

Uncontrolled pain can cause cardiovascular instability, respiratory compromise and immunosuppression in the critically ill.^139^ Analgesia should be prioritized during initial resuscitation of septic dogs and cats (Figure 1). Due to pain severity, opioids are commonly used in SP, but multimodal therapy may provide superior analgesia with fewer adverse effects. Non‐steroidal anti‐inflammatory drugs (NSAIDs) should be avoided due to the risk of renal injury and gastric ulceration.^140^

Ketamine and lidocaine CRIs reduce opioid requirements.^141^ Lidocaine also has anti‐inflammatory free radical scavenging and antiarrhythmic properties.^142^, ^143^ Intraoperative opioid plus lidocaine CRI improved short term survival over opioid alone in dogs with SP.^20^ Within the smaller subset of patients that received lidocaine CRI postoperatively, there was increased mortality, which could not be explained by variables examined in the study.^20^ Lidocaine CRIs should not be administered to feline patients due to a narrow safety margin.

Dexmedetomidine provides analgesia and anxiolysis, but decreases systolic function and cardiac output.^144^ Dexmedetomidine may improve vascular reactivity in sepsis by downregulating catecholamines and resensitizing alpha‐1 receptors.^145^ The Sedation Practice in Intensive Care Evaluation (SPICE III) trial in critically ill people suggested lower vasopressor requirements in patients receiving dexmedetomidine,^146^ but a pilot study in people with refractory septic shock was stopped early due to increased mortality.^147^ A pilot study of intraoperative low‐dose dexmedetomidine infusion in septic dogs demonstrated decreased vasopressor requirement and improved 28 day survival;^148^ however, a similar study found no benefit.^149^ Further research is needed before dexmedetomidine can be recommended for routine use in septic small animals.

## 6 ANESTHESIA

### 6.1 Fluid therapy

Prior to anesthetic induction and throughout anesthesia, hemodynamic optimization should be pursued, including transfusion as indicated.^64^ Once intravascular volume has been restored, aggressive fluid resuscitation should cease. Fluid elimination decreases with hypotension and during anesthesia. While this generally obviates the need for large volumes of fluids during surgery,^55^, ^150^ surgical losses and uncorrected volume deficits should be replaced.^106^

### 6.2 Premedication and anesthetic induction

Premedication is often accomplished with the analgesics described above, and may include a benzodiazepine for additional sedation and muscle relaxation with minimal cardiovascular effects.^151^ Induction strategy should prioritize rapid intubation. Preoxygenation should be performed, and maropitant should be considered to minimize aspiration risk.^152^ Patients in shock will usually be more sensitive to hemodynamic and neurologic effects of anesthetic agents and may require lower doses.^153^ Selection and titration of induction agent(s) and anesthetic maintenance is performed with a goal of minimizing further impairment of hemodynamics. Selected induction agents are highlighted in Table 2.

**TABLE 2:** Selected induction agents for septic dogs and cats.

Drug	Advantages	Disadvantages	Additional considerations
Ketamine	Cardiac output, heart rate, blood pressure, and respiratory drive preserved^64^, ^153^	May cause tachycardia^64^ May increase intracranial pressure^64^	Sympathomimetic properties may be blunted with catecholamine insensitivity^64^
Propofol	Rapid, smooth induction^154^	Vasodilation^154^ Myocardial depression^154^ Respiratory depression^154^	Titrate slowly to lowest effective dose^155^ Combine with ketamine to mitigate cardiovascular depression^155^
Alfaxalone	Rapidly metabolized^155^ Wide safety margin^155^	Modest cardiorespiratory depression similar to diazepam/fentanyl induction^155^	Excitement on recovery^64^
Fentanyl/Midazolam	Minimal cardiovascular effects^155^	Respiratory depression^155^ Increased vagal tone at high doses^155^	Consider anticholinergics if clinically important bradycardia occurs^156^ Benzodiazepines improve muscular relaxation^64^ Midazolam is highly protein bound; reduce dose with severe hypoalbuminemia^64^
Etomidate	Minimal cardiovascular and respiratory effects^157^	Adrenocortical suppression^158^	Associated with poorer outcomes in critically ill, septic people^64^, ^153^, ^157^, ^158^, ^159^

### 6.3 Local analgesia

Local blocks can provide analgesia intraoperatively and facilitate postoperative pain management. Reports are mixed on the efficacy of liposome‐encapsulated bupivacaine (LEB) for canine celiotomy incisional blocks.^160^, ^161^, ^162^ Ultrasound‐guided transverse abdominis plane (TAP) blocks and rectus sheath (RS) LEB blocks in dogs appeared to offer adequate intra‐ and postoperative analgesia,^160^ while ultrasound‐guided RS blocks in cats did not.^163^ Incisional LEB blocks did not increase the risk of surgical site infection in dogs with SP.^164^ When patient stability permits, and trained personnel that can rapidly perform these blocks are available, TAP and RS blocks should be considered for dogs with SP.

### 6.4 Maintenance of anesthesia

Maintenance of anesthesia is achieved with volatile anesthetics, intravenous anesthetics, or both. Inhaled anesthetics, such as isoflurane and sevoflurane, cause dose‐dependent vasodilation and myocardial depression, but may have beneficial immune‐modulating effects.^165^, ^166^ Adjunctive CRIs of propofol, alfaxalone, opioids, ketamine, or benzodiazepines lessen hemodynamic impacts of inhaled anesthetics.^152^ Higher doses of injectable anesthetics allow total intravenous anesthesia, which reduces postoperative nausea and vomiting in people.^167^ Patients experiencing hypoventilation or with respiratory compromise may require mechanical ventilation.^168^

### 6.5 Management of intraoperative hypotension

Intraoperative hypotension is common in septic patients,^169^ and has been associated with increased risk of intestinal dehiscence in dogs,^170^ and higher mortality in dogs with SP, particularly if prolonged.^31^, ^35^, ^171^ Blood pressure should be monitored closely and hypotension promptly corrected. Inhaled and intravenous anesthetics should be titrated to avoid excessive anesthetic depth, and heat support should be provided.^106^ Additional volume resuscitation may be necessary.

Dynamic measures of fluid responsiveness are preferred for assessing intravascular volume. Limited access to the patient hampers the use of ultrasound‐derived variables, however arterial waveform analysis may be helpful. In mechanically ventilated dogs with stable tidal volume and a normal right heart, pulse pressure variation (PPV) predicted fluid responsiveness with 78.6%–100% sensitivity and 80%–100% specificity.^172^, ^173^, ^174^ Pulse oximeter waveform analysis yields the pleth variability index (PVI), which predicted volume responsiveness with 85.71% sensitivity and 70% specificity in an experimental canine study.^172^ PVI‐guided fluid therapy reduced incidence of hypotension and lowered total fluid volume given in American Society of Anesthesiologists (ASA) Physical Status Classification System 1–2 dogs.^175^

When PPV or PVI measurement is not feasible, preoperative fluid‐responsiveness and serial packed cell volume (PCV) can help guide intraoperative fluid administration. A fluid challenge provides further insight. Septic patients may be non‐responsive to fluid therapy due to vasoplegia or myocardial dysfunction, side effects of anesthetic agents, or anesthetic depth. Regardless of the cause, patients non‐responsive to fluids should receive vasoactive therapy as described above.^60^ Patients with reduced systolic function or persistent hypoperfusion despite afterload optimization with pressor therapy should receive dobutamine.^59^, ^64^

## 7 POSTOPERATIVE MANAGEMENT

Intensive nursing care is required in the postoperative period and has been thoroughly reviewed elsewhere.^176^ Immediate postoperative management involves continued optimization of hemodynamic and oncotic status, fluid balance, oxygen carrying capacity, coagulation, pressor support, analgesia, antibiotic stewardship, management of abdominal drains and nutritional support.

### 7.1 Nutrition

Most veterinary patients with SP are ill for >3 days, putting them at high risk for malnutrition.^177^ Preoperative nutritional assessment allows planning for intraoperative feeding tube placement.^177^ Short‐term enteral nutrition (EN) can be delivered through nasoesophageal or nasogastric tubes, while long‐term nutrition requires an esophagostomy or gastrostomy tube. Feedings should be gradually increased to meet resting energy requirements within 3 days, if tolerated.^178^, ^179^ In patients managed with an open abdomen, protein losses and repeated sedation or anesthesia are expected, and the nutritional plan must consider any preanesthetic fasts.

Parenteral nutrition should be reserved for when EN is contraindicated or not tolerated despite pharmacologic therapy. The incidence of metabolic complications is high,^180^ and although rare, septic complications can be life‐threatening.^181^ Practical considerations, indications and complications are reviewed elsewhere.^177^

In people, EN is recommended within 72 h of sepsis diagnosis.^52^, ^179^ Enteral nutrition protects enterocytes, mitigates abnormal gut permeability, improves motility, and may prevent organ failure.^182^, ^183^, ^184^ Enteral feeding causes splanchnic vasodilation, so EN should be delayed until cardiovascular stability is achieved.^182^, ^184^ The use of vasopressors does not preclude EN in volume‐replete patients with adequate MAP on stable pressor doses.^182^

Early EN (<48 h postoperatively) resulted in fewer pulmonary complications, shorter hospitalization time, and reduced mortality in people undergoing emergency gastrointestinal surgery,^185^ and EN within 24 h postoperatively decreased hospitalization for lower gastrointestinal surgery.^186^ Retrospective veterinary studies offer limited and inconsistent data on the impact of EN in SP, but major complications appear to be uncommon (Table 3).^19^, ^28^, ^29^, ^187^, ^188^ Given the documented benefits of EN in people, and the low incidence of major complications in veterinary species, early EN should be instituted in dogs and cats unless contraindicated.

**TABLE 3:** Retrospective studies of enteral and parenteral nutrition in dogs after surgery for septic peritonitis.

Year	Population (n)	Route	Complications	Timing	Effect on outcome
**2020** ^28^	GT placed at time of surgery (43)	GT	Discharge from tube site: 10 Premature removal by patient: 1 Breakage of tube: 1	Mean time to first food intake 16+/−5 h	Lower complication rate than previously reported with other indications for GT
**2019** ^187^	Surviving >48 h (68)	EN Voluntary intake: 23 Feeding tube only: 7 PN only: 10 PN + EN: 28 38 feeding tubes: 23 NG 8 NE 6 Gastrostomy 1 Duodenostomy	Tube dislodgement: 7/36 Vomiting or regurgitation: 12/36 (NG>NE) PN catheter dislodgement: 2/38	Median 1 day for all groups	No association between time until nutrition and survival PN associated with non‐survival No benefit of PN + EN over EN alone
**2019** ^29^	GT placed at time of surgery (28)	GT	Vomiting, regurgitation, or increased gastric residual volume: 4 Peristomal inflammation: 4 Patient dislodgement: 2 Subcutaneous migration requiring removal: 2	Median time to feeding 1 day (0–2)	Time until feeding associated with LOH (*p* = .036)
**2017** ^19^	Surviving >24 h (65)	16/21 (76.2%) NG or NE 2 jejunostomy 3 GT	Suspected aspiration pneumonia: 2 1 Early 1 No nutrition	<24 h: 16 >24 h: 27 None: 13	Dogs that received any nutrition more likely to survive
**2012** ^180^	Survivors (45)	Voluntary intake: 31 GT: 1 (4 placed; only 1 used for nutrition) PN: 13	PN catheter site infection:1 Gastric leakage around GT, requiring surgery: 2 Leakage from PN catheter requiring removal: 1	<24 h: 12 >24 h: 33	Decreased LOH with early nutrition (<24 h)

Abbreviations: EN, enteral nutrition; GT, gastrostomy tube; LOH, length of hospitalization; NE, nasoesophageal tube; NG, nasogastric tube; PN, parenteral nutrition; SP, septic peritonitis.

### 7.2 Thromboprophylaxis

Although dogs with SP are often hypercoagulable,^189^ the American College of Veterinary Emergency and Critical Care Consensus on the Rational Use of Antithrombotics in Veterinary Critical Care guidelines^190^ found insufficient evidence to recommend routine thromboprophylaxis. Antithrombotics are recommended in dogs with SP only when hypercoagulability is demonstrated or additional risk factors are present,^190^ and in cats only when risk factors for thrombosis exist, or after assessment of the risks/benefits in individual patients.^191^ Although not specifically recommended in SP, direct oral anticoagulants or low molecular weight heparins are suggested for venous thrombosis,^190^ the most likely thromboembolic complication of sepsis.^192^

## 8 COMPLICATIONS

In recent years, survival rates for dogs and cats having surgery for SP range from 36.4% to 100% regardless of the source of SP,^13^, ^16^, ^17^, ^18^, ^19^, ^20^, ^21^, ^22^, ^23^, ^24^, ^25^, ^26^, ^27^, ^28^, ^29^, ^30^, ^31^, ^32^, ^33^, ^34^, ^35^, ^36^, ^37^, ^193^, ^194^ These numbers span wide ranges, in part due to limitations of retrospective studies and the confounder of euthanasia, but mortality remains substantial. Complications that contribute to mortality include multiple organ dysfunction, coagulopathy, fluid overload, recurrent SP, hospital acquired infections, and intra‐abdominal hypertension.

### 8.1 Recurrent septic peritonitis

Recurrent SP (RecSP) is SP that occurs after surgery for SP. In veterinary studies with cases extending into the last 15 years, RecSP survival rates ranged from 0%–43.9% (Table 1).^16^, ^22^, ^23^, ^170^, ^195^, ^196^, ^197^, ^198^, ^199^ The most common cause appears to be dehiscence of a gastrointestinal surgery site. Dehiscence rates of intestinal anastomoses were higher in dogs with preoperative SP (6.4%–33%) than without (1.8%–11.5%).^195^, ^196^, ^197^, ^198^, ^200^, ^201^, ^202^, ^203^ Common components of SP that adversely affect gastrointestinal healing include hypoperfusion, hypovolemia, impaired collagen synthesis, excessive collagenases and inflammatory cytokines, microbial dysbiosis, and malnutrition.^204^, ^205^, ^206^, ^207^ While albumin plays direct and indirect roles in healing, preoperative hypoalbuminemia in dogs and cats with SP was associated with increased risk of gastrointestinal dehiscence in some studies^22^, ^208^, ^209^, ^210^ but not others.^25^, ^71^, ^195^, ^197^, ^198^, ^200^, ^201^, ^203^, ^211^, ^212^, ^213^, ^214^ Intraoperative hypotension inconsistently correlated with dehiscence in older studies,^22^, ^201^ while hypotension during hospitalization correlated with dehiscence in a recent retrospective study.^195^ While having a foreign body as the reason for anastomosis was associated with anastomotic leakage in an older review,^210^ this was not found in recent studies.^16^, ^22^, ^195^, ^198^, ^202^, ^205^, ^208^

Any deterioration in the patient's postoperative clinical condition or clinicopathologic variables should prompt a search for recurrent SP. Unfortunately, short of exploratory laparotomy, definitive diagnosis of recurrent SP is difficult.^8^ Postoperative abdominal fluid analysis is challenging as the usual markers of SP no longer hold true. Cell counts and presence of bacteria do not predict intestinal dehiscence.^169^, ^215^ Additionally, bacteria and whole blood‐effusion glucose (WB‐EGD) and lactate differences (WB‐ELD) do not distinguish septic from non‐septic effusions collected from abdominal drains.^169^, ^215^

In dogs with peritoneal drains, degenerate neutrophils and intracellular bacteria should be expected. Cytology of abdominal fluid from healthy dogs undergoing midline celiotomy, peritoneal lavage, and drain placement had predominantly degenerate neutrophils, with bacteria observed in 40%.^215^ All dogs with ongoing drain production had WB‐EGD >20 by day 4, and 7/8 had WB‐ELD <−2 by day 4. Although individual effusion lactate values were not reported, by day 3, mean lactate was higher than the 4.2 mmol/L cutoff reported by Martiny et al. for diagnosing canine SP. ^216^ When dogs with uncomplicated recovery from gastrointestinal surgery and closed suction drain placement for SP were retrospectively compared to those that developed RecSP, WB‐EGD and WB‐ELD failed to predict or identify patients that developed RecSP.^23^ Bacteria and degenerate neutrophils were present in abdominal fluid of patients with uncomplicated recoveries. Bacteria were not identified in the effusion of any RecSP dogs, and neither the effusion white blood cell count (WBC) nor the volume of peritoneal effusion increased at the time of RecSP development. In fact, 2/3 dogs with RecSP had effusion WBC <500/μL.

There are few reports describing postoperative fluid analysis in dogs undergoing resection and anastomosis without drain placement, and to the authors' knowledge, there are none specifically evaluating postoperative fluid in dogs that had preoperative SP. One study of dogs recovering from resection and anastomosis reported fluid analysis findings in dogs with and without drain placement at 24 and 48 h, and found no significant difference in effusion glucose, EB‐EGD, effusion lactate or WB‐ELD in those who developed intestinal dehiscence.^199^ Bacteria were detected in the effusion of four patients at 24 h, only two of which went on to develop intestinal dehiscence, although it was not reported whether these patients had abdominal drains in place.^199^ Of those dogs that developed intestinal dehiscence, 3/5 had no bacteria seen on cytology.^199^

Postoperative ultrasound evaluation of the intestinal surgical site and surrounding tissues by trained ultrasonographers shows promise as a sensitive and specific tool to detect early dehiscence.^208^ Ultimately, diagnostic tools are limited and clinicians must rely on their interpretation of their patient's clinical progression in light of risk factors for surgical site dehiscence and time since surgical intervention to decide if re‐exploration is warranted. When in doubt, clinicians should err on the side of re‐exploration.

### 8.2 Hospital acquired infection

Hospital acquired infections occur frequently in small animal ICUs.^217^ Surgical site infections, hospital acquired pneumonia, and device‐related infections occur. Incisions, drain exits, and catheter sites should be examined daily. Blood stream infections (BSI) are rare in veterinary patients, although IV catheter colonization is common. Risk factors include dextrose infusion, longer duration of catheterization, and immunocompromise.^218^ Prophylactic IV catheter replacement does not decrease BSI risk in people, and is not recommended in animals.^217^ Catheter‐associated urinary tract infections are reported in 10.3%–55% of catheterized dogs, with risk factors including longer catheterization time, increasing age, and concurrent antimicrobial administration.^219^, ^220^, ^221^ Ultrasonographic estimation of urine volume may provide a useful surrogate for urine quantification without the need for indwelling urethral catheterization;^222^ however, indwelling urethral catheterization facilitates patient cleanliness and monitoring for intra‐abdominal hypertension.

### 8.3 Intra‐abdominal hypertension

Intra‐abdominal hypertension (IAH, defined as intraabdominal pressure (IAP) ≥ 12 mmHg) is estimated to occur in 50%–80% of critically ill people.^223^ In people with SP due to GI perforation, preoperative IAH was present in 28% (if non‐traumatic cause) to 66% (traumatic or non‐traumatic).^224^, ^225^ Postoperative IAH was present in 12% to 41% of SP patients in the first 24 h, and correlated with prolonged ileus, postoperative peritonitis, organ dysfunction, and mortality.^224^, ^225^, ^226^

Factors contributing to IAH in SP patients include free fluid, bowel distension from edema and ileus, abdominal muscle contraction guarding against pain, and aggressive fluid management.^11^, ^225^, ^226^, ^227^, ^228^, ^229^ Intrabdominal hypertension compromises respiration and visceral perfusion and can result in bacterial translocation, compromised healing, organ dysfunction, and death.^11^, ^224^, ^227^, ^228^

Increasing recognition of IAH has led to more vigilant monitoring in human ICUs.^223^, ^230^ In veterinary medicine, the potential impact of IAH in patients with SP or other critical illness has generally been overlooked. A transurethral catheter or nasogastric tube, commonly placed in critical veterinary patients, allow IAP measurement.^228^ Normal canine IAP is 1.5–5.2 mmHg; IAP >20 mmHg warrants treatment.^228^ Treatments include abdominal decompression via abdominocentesis or nasogastric or rectal tube, relaxation with neuromuscular blockade and sedation, adequate analgesia, and open abdomen management.^11^, ^227^, ^228^, ^231^ Monitoring IAP in at risk patients may lead to earlier intervention and improved outcomes.

### 8.4 Mortality

Table 1 shows survival data on canine and feline SP patients treated surgically. The included papers all had patient populations extending into the last 15 years (i.e., at least to 2010). They are ordered by the span of years covered instead of publication date to better represent the relative time of treatment. Survival rates when all study years were 2010 to present^23^, ^24^, ^26^, ^27^, ^30^, ^34^, ^37^, ^170^ trended higher (most >75%) than when the preponderance of study years were before 2010 (most <67%),^16^, ^17^, ^18^, ^19^, ^22^ suggesting improvements in care. When assessed, survivors had lower disease severity,^18^, ^30^, ^32^, ^36^ less hypotension,^31^, ^32^ and earlier nutrition than non‐survivors. Unsurprisingly, survival rates were consistently lower (0%–43.9%) in patients with recurrent SP.^16^, ^22^, ^23^, ^170^ The impact of appropriate empirical antibiotics on survival was mixed, as discussed above.^13^, ^16^, ^18^, ^27^, ^31^, ^34^ The impact of open versus closed abdomen +/−drainage is difficult to assess and discussed further in a companion study.^8^

Despite the information provided, Table  illustrates the preponderance of retrospective studies and variations in inclusion criteria, data recorded, and treatments assessed that make it difficult to discern best practices for improving SP survival in veterinary patients.

## 9 SUMMARY

Septic peritonitis is driven by complex pathophysiological changes. Although evidence to guide treatment is limited by the retrospective nature of most studies and diverse patient populations, some conclusions can be drawn. Based on the available human and veterinary literature, initial crystalloid bolus followed by careful titration of fluid therapy, and early intervention to include adequate analgesia, appropriate antimicrobial therapy and postoperative enteral nutrition are recommended. Gaps exist in the understanding of the ideal resuscitation strategy, antimicrobial selection and timing, anesthetic approach, and especially in the accurate diagnosis of recurrent SP. Further research in dogs and cats with septic peritonitis exploring the role of natural colloids in volume resuscitation, potential benefits of including lidocaine or alpha‐2 agonists in the anesthetic protocol, the impact of volatile anesthetics on the immune response, risks or benefits of thromboprophylaxis, and incidence and impact of IAH are indicated to refine perioperative care of small animals with SP and improve outcomes.

## AUTHOR CONTRIBUTIONS

O'Marra SK, DVM, DACVECC and Campbell BG, DVM, PhD, DACVS: Collaborated on the design of the manuscript. Each author wrote substantial portions of the document, critically reviewed the manuscript, and endorsed the final version. The authors are aware of their respective contributions and have confidence in the integrity of all contributions.

## FUNDING INFORMATION

No funding was received for this manuscript.

## CONFLICT OF INTEREST STATEMENT

The authors declare that they have no conflicts of interest relevant to this manuscript.
